# Rapid assessment of case recruitment tools to inform integrated surveillance of influenza and other respiratory viruses in Eastern Mediterranean countries

**DOI:** 10.1111/irv.13132

**Published:** 2023-04-23

**Authors:** Erin Mathieu, Noore Alam, Hala Abou El Naja, Wasiq Khan

**Affiliations:** ^1^ World Health Organization Regional Office for the Eastern Mediterranean Cairo Egypt; ^2^ Sydney School of Public Health University of Sydney Camperdown New South Wales Australia

**Keywords:** influenza, sentinel surveillance, World Health Organization

## Abstract

Influenza‐like illness (ILI) and severe acute respiratory infection (SARI) case recruitment tools from 10 countries were reviewed. The contents of the existing tools were compared against World Health Organization's current guidelines, and we also assessed the content validity (accuracy, completeness and consistency). Five of the ILI tools and two of the SARI tools were rated as having high accuracy against WHO case definitions. ILI completeness ranged from 25% to 86% and SARI from 52% to 96%. Average internal consistency scores were 86% for ILI and 94% for SARI. Limitations in the content validity of influenza case recruitment tools may compromise recruitment of eligible cases and result in varying detection rates across countries.

## 1 BACKGROUND

Influenza is an acute respiratory infection caused by influenza viruses that circulate in all parts of the world. Illnesses range from mild to severe and even death. Hospitalisation and death occur mainly among high risk groups. Worldwide, these annual epidemics are estimated to result in about 3 to 6.5 million seasonal INFLUENZA‐associated deaths (4.0–8.8 per 100,000 individuals).^1^

For over 70 years, influenza sentinel surveillance systems have been used globally to detect influenza epidemics and assess seasonal patterns and severity in a timely manner for implementing effective management strategies and interventions.^2^

The WHO's Global Influenza Programme provides guidance for influenza surveillance.^3^ The WHO Regional Office for the Eastern Mediterranean provides operational and technical support for countries in the Eastern Mediterranean Region (EMR) to assist with evidence‐informed surveillance practices including the provision of robust tools for accurate and timely detection of influenza and other respiratory pathogens of epidemic and/or pandemic potential.^4^

For the purpose of valid data collection and reporting, case recruitment tools used at influenza sentinel surveillance sites need to be robust and methodologically sound to ensure valid case selection.

## 2 AIM

The aim of this rapid assessment exercise was to review existing and available case recruiting tools for influenza‐like illnesses (ILI) and severe acute respiratory infections (SARI) from selected countries and assess their content validity.

## 3 METHODS

ILI and SARI case recruitment tools from 10 countries in EMR were reviewed (Afghanistan, Bahrain, Jordan, Lebanon, Libya, Morocco, Saudi Arabia, Somalia, Sudan and Yemen). The content of each country's tool was compared against WHO guidelines^2^ and Eastern Mediterranean regional influenza data reporting platform, EMFLU‐2.0.^5^ Content validity, measuredas (i) accuracy of case definitions, (ii) completeness of needed variables and (iii) consistency and usability of recruitment tools, were assessed.

1. **Accuracy** was assessed against the current WHO case definition for ILI (an acute respiratory infection with measured fever greater than or equal to 38°C and cough with onset within the last 10 days) and SARI (an acute respiratory infection with measured fever greater than or equal to 38°C and cough with onset within the last 10 days and requires hospitalisation).^1^ Two elements were considered in assessing accuracy: (i) statement of case definition criteria listed and (ii) questions eliciting attributes of case definition criteria embedded within the tool which allow for either identification or exclusion of the patient as a case. The combination of these two elements determined the overall accuracy using the matrix below (Table 1).
2. **Completeness** of variables listed in the country's tool was assessed against the current reference standard (EMFLU‐2), which is aligned to the global epidemiological surveillance standards for influenza,^2^ by calculating proportion agreement for Member States' against the EMFLU‐2 variable list. Agreement was assessed as the overall number of variables in agreement in each tool as a proportion of the total number in EMFLU‐2 (*N* = 41 for ILI and *N* = 52 for SARI). Results are presented on a continuous scale ranging from 0% to 100%, with 100% indicating complete agreement with EMFLU‐2.
3. **Consistency and usability** were assessed using a newly developed assessment tool which considered the following: (i) clarity (defined as clear/unambiguous information or question wording); (ii) consistency of answer fields in the tool (similar scales, ease of recording responses thereby reducing the chance of errors); (iii) logical flow (taking into account the natural progression of a patient interview/assessment); (iv) length of the tool (too long or too short). Each element was rated independently by an expert on a scale of 1–3 (with 3 *being the most desired level of element* and 1 *being the least desired*), and the overall consistency and usability was converted to a scale of 0–100* with higher scores indicating higher levels of consistency and usability.

**TABLE 1:** Required criteria elements measuring ‘accuracy’ of SARI/ILI case definitions embedded in the tool.

		Attributes of case definition criteria embedded in the tool		
Statement of case definition criteria requirements		Criteria are correct and completely embedded^a^ and it is clear that questions met the case definition criteria	All key criteria are contained in the tool, but not all are embedded^a^	The tool does not contain sufficient questions to determine if criteria are met
	Criteria are complete and correct	A ‐ High	C ‐ Medium	E ‐ Low
	Criteria are not listed	A ‐ High	C ‐ Medium	F ‐ Low
	Criteria are listed, but are incomplete or incorrect	B (due to conflicting/contradictory information) ‐ Medium	D (due to conflicting/contradictory information) ‐ Medium	F ‐ Low

^a^ Embedded – Key questions to determine if case definition is met are presented as a group with clear recognition that these group of questions are used to determine if a patient meets the criteria of being a potential case of SARI or ILI.

## 4 RESULTS

The ILI case recruitment tools for 10 countries and the SARI case recruitment tools for nine countries were reviewed. Results are summarised in Table 2.

**TABLE 2:** Accuracy, completeness and consistency of ILI and SARI case recruitment tools.

Country	ILI case recruitment tools					SARI case recruitment tools				
	Accuracy^a^		Completeness		Consistency/usability	Accuracy^a^		Completeness		Consistency/usability
	Rating	Level	# variables in tool	% completeness	Score (0–100)	Rating	Level	# variables in tool	% completeness	Score (0–100)
Afghanistan	C	Medium	23	37	88	F	Low	42	58	88
Bahrain	C	Medium	26	20	88	A	High	40	63	100
Jordan	A	High	29	59	75	C	Medium	40	65	75
Lebanon	A	High	28	29	75	C	Medium	41	44	100
Libya	A	High	41	95	100	A	High	52	94	100
Morocco	C	Medium	49	59	88	C	Medium	55	58	88
Saudi Arabia	A	High	42	93	100	C	Medium	50	92	100
Somalia	A	High	39	95	100	C	Medium	49	92	100
Sudan	C	Medium	32	44	63	C	Medium	52	44	75
Yemen	D	Low	45	41	75	NA^b^	NA^b^	NA^b^	NA^b^	NA^b^

^a^ Refer to Table 1 for descriptions.

^b^ NA, not available. Yemen did not provide their SARI case recruitment tool for inclusion in this evaluation.

### 4.1 Accuracy (case definition)

As seen in Table 2, five of the 10 ILI tools were rated as having high levels of accuracy (Jordan, Lebanon, Libya, Saudi Arabia and Somalia). These tools contained sufficient information in a clear way to support the purpose and meaning of case definition. Yemen's tool incorrectly noted the fever component of the case definition (greater than 38°C), which resulted in them being classified as ‘Low’.

Only two of the SARI tools were classified as having high levels of accuracy (Bahrain and Libya). Six tools (Jordan, Lebanon, Morocco, Saudi Arabia, Somalia and Sudan) received a ‘Medium’ rating due to not listing the case definition and having incomplete case definition question embedded within their tool. Afghanistan received a ‘Low’ rating as they did not note whether the case had a cough, an essential element of case definition for both SARI and ILI.

### 4.2 Completeness

**FIGURE 1:**
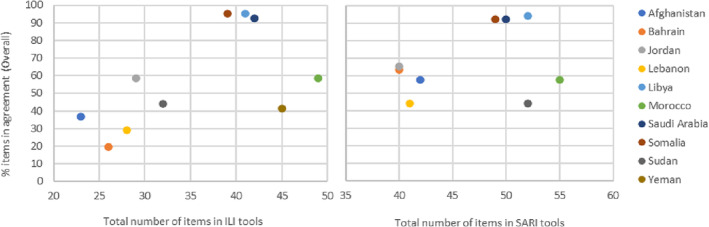
Agreement (%) of Member Countries' ILI and SARI tools compared with EMFLU‐2 database.

The total number of variables contained in ILI tools ranged from 23 variables (Afghanistan) to 49 variables (Morocco) with a median of 35.5 variables. However, the total number of variables in agreement with the EMFLU‐2 database (a possible 41 variables) was as low as 8 (20%) in Bahrain to as high as 39 (95%) in both Libya and Somalia. Having a tool with more variables did not equate to having higher levels of agreement with EMFLU‐2 (Figure 1).

The total number of variables in SARI tools ranged from 40 (Bahrain and Jordan) to 55 variables (Morocco) (Median = 49 variables). When compared with the EMFLU‐2 database (a possible 52 variables), Lebanon and Sudan had the lowest levels of overall agreement at 44% with Libya, Somalia and Saudi Arabia reaching agreement levels of over 90% (Table 2). Longer and shorter tools had lower agreement compared with those closer to the median number of variables (Figure 1).

### 4.3 Consistency and usability

Across the board, tools were relatively consistent and usable with high levels of clarity in question wording, consistent answers, a logical flow and an appropriate length. ILI scores ranged from 62.5 (Sudan) to 100 (Libya, Saudi Arabia and Somalia), with an overall average score of 85. SARI tools scored a little higher, with scores ranging from 75 (Sudan and Jordan) to 100 (Bahrain, Lebanon, Libya, Saudi Arabia and Somalia), with an overall average score of 92. Improvements could be made in the logical flow of the tools for some of the counties.

## 5 DISCUSSION

This rapid assessment has identified some areas of improvement in the identification of cases and the collection of data in some of the existing case recruitment forms.

Case identification was not apparent, nor readily available in many of the case recruitment tools. The lack of this may introduce selection bias in identifying cases when they present with symptoms at the sentinel sites, especially, if staff is not well trained or where there is high staff turnover. Some tools included the case identification questions further down the tool. This is less than ideal, potentially resulting in the reporting of false positive cases if not carefully rechecked at the end (i.e., forms are completed or not; however, it is later identified that the case does not present required symptoms to meet the case definition). This potentially results in staff at sentential sites spending unnecessary time completing case recruitment forms and associated administrative work to generate false positive cases.

Various tools with different levels of accuracy, completeness and consistency are insufficient to collect the most pertinent information on influenza cases and to meet global reporting requirements, making comparison between countries within the region and globally a challenge. Robust tools reflect WHO's ILI and SARI case definitions, and a balance between length of the tool and sufficient data capturing is essential. A tool with fewer questions may miss important variables, and a tool with too many questions, because of demanding schedules of clinicians, may lead to partial completion of the forms, resulting in higher levels of missing data. Results of this assessment indicate that it is possible to have high levels of accuracy, completeness (when compared with the reference standard), and consistency without having an overly long tool. It is, however, important to note that three of the countries with higher levels of content validity (Libya, Saudi Arabia and Somalia) adapted the EMFLU2 form while implementing their surveillance system, which would explain their high scores.

There is a clear overlap in the variables contained within ILI and SARI case recruitment tools. Despite this, several countries used different tools, with varying levels of content validity. To improve the quality of surveillance outputs within and across countries especially in the context of integrated surveillance for influenza and respiratory pathogens of epidemic and pandemic potential, it is recommended that countries agree on a minimum set of variables to be included in case recruitment tools enabling valid comparisons to be made across countries.

### 5.1 Limitations

There are several limitations in the conduct of this rapid assessment. Firstly, EMFLU‐2 was used as the reference standard, rather than a gold standard. Although it was developed in accordance with WHO requirements, it may not contain all required variables and may contain additional variables not required for mandatory reporting. It was selected as a means to make a standardised comparisons across all countries included in the review.

Regardless of how robust the tool may be, it does not necessarily translate into accurate identification and recruitment of cases in the field. Along with quality tools, it is also important to have adequate and regular training of the sentinel sites staff. Furthermore, it is also important to have periodic monitoring and evaluation exercises including verification/validation of a proportion of completed tools to check for the validity of surveillance outputs.

The methods used in the evaluation of accuracy and consistency of the tools were developed specifically for this evaluation. They were developed with explicit criteria and standards in an attempt to minimise subjectivity as much as possible. They are not standardised or validated techniques and, hence, should be used keeping this in mind. Our results, therefore, should be interpreted taking this into consideration.

Attempts were made to obtain ILI and SARI case recruitment tools from all EMR countries. Unfortunately, only 10 countries were able to provide their tools within the timeframe of the evaluation. It is possible that the 10 countries that supplied their tools are representative of all countries in the region; however, this is not possible to assess. As such, other countries in the region may have case reporting tools that rate higher or lower than the case reporting tools evaluated in this study.

## 6 CONCLUSION

Limitations in accuracy, completeness and consistency of the influenza case recruitment tools may compromise recruitment of eligible cases for influenza testing which may result in low or varying detection rates across countries. In the context of WHO's roadmap to multi respiratory pathogens integrated surveillance,^6^, ^7^ the development of an integrated tool which contains a minimum set of variables that meets the WHO global standards for integrated surveillance would potentially allow for standardisation of data collection across countries in the EMR. Further evaluation, including testing the integrated tool for reliability and validity and pilot testing for usability by the influenza focal points in countries, should be undertaken prior to wide‐spread implementation of a new integrated recruitment tool.

## AUTHOR CONTRIBUTIONS

**Erin Mathieu:** Data curation; formal analysis; investigation; methodology; project administration; writing—original draft; writing—review and editing. **Noore Alam:** Conceptualization; formal analysis; methodology; supervision; writing—review and editing. **Hala Abou El Naja:** Data curation; investigation; methodology; project administration; writing—review and editing. **Wasiq Khan:** Conceptualization; funding acquisition; writing—review and editing.

## CONFLICT OF INTEREST STATEMENT

All authors note that there are no conflicts of interest to report.

### PEER REVIEW

The peer review history for this article is available at https://www.webofscience.com/api/gateway/wos/peer-review/10.1111/irv.13132.

## Data Availability

The data that support the findings of this study are available from the corresponding author upon reasonable request.

## Footnotes

* Overall score = $\frac{\sum_{1}^{4}s_{n}-1}{8}\times 100$ where s = score for each element (elements 1–4) with each score ranging from 1–3.
